# Lessons from a GWAS study of a wheat pre-breeding program: pyramiding resistance alleles to Fusarium crown rot

**DOI:** 10.1007/s00122-020-03740-8

**Published:** 2020-12-26

**Authors:** Marcos Malosetti, Laura B. Zwep, Kerrie Forrest, Fred A. van Eeuwijk, Mark Dieters

**Affiliations:** 1 Mathematical and Statistical Methods (Biometris), Wageningen University and Research, Wageningen, The Netherlands; 2 Mathematical Institute, Leiden University, Leiden, The Netherlands; 3 Agriculture Victoria Research, Agribio, Bundoora, Melbourne, VIC 3083 Australia; 4 School of Agriculture and Food Sciences, Faculty of Science, The University of Queensland, Brisbane, Australia

## Abstract

Much has been published on QTL detection for complex traits using bi-parental and multi-parental crosses (linkage analysis) or diversity panels (GWAS studies). While successful for detection, transferability of results to real applications has proven more difficult. Here, we combined a QTL detection approach using a pre-breeding populations which utilized intensive phenotypic selection for the target trait across multiple plant generations, combined with rapid generation turnover (i.e. “speed breeding”) to allow cycling of multiple plant generations each year. The reasoning is that QTL mapping information would complement the selection process by identifying the genome regions under selection within the relevant germplasm. Questions to answer were the location of the genomic regions determining response to selection and the origin of the favourable alleles within the pedigree. We used data from a pre-breeding program that aimed at pyramiding different resistance sources to Fusarium crown rot into elite (but susceptible) wheat backgrounds. The population resulted from a complex backcrossing scheme involving multiple resistance donors and multiple elite backgrounds, akin to a MAGIC population (985 genotypes in total, with founders, and two major offspring layers within the pedigree). A significant increase in the resistance level was observed (i.e. a positive response to selection) after the selection process, and 17 regions significantly associated with that response were identified using a GWAS approach. Those regions included known QTL as well as potentially novel regions contributing resistance to Fusarium crown rot. In addition, we were able to trace back the sources of the favourable alleles for each QTL. We demonstrate that QTL detection using breeding populations under selection for the target trait can identify QTL controlling the target trait and that the frequency of the favourable alleles was increased as a response to selection, thereby validating the QTL detected. This is a valuable opportunistic approach that can provide QTL information that is more easily transferred to breeding applications.

## Introduction

Fusarium crown rot (FCR) is an important disease in wheat caused primarily by the pathogen *Fusarium pseudograminearum*. In Australia, the incidence and severity of FCR has increased; that is, the disease has been observed more frequently across years and when detected a larger proportion of the crop area is affected. Estimated losses due to FCR are in the order of AUS$80 million, with the estimated losses of up to 10–20% of the total crop production (Murray and Brennan 2009). Cultural practices such as crop rotation and elimination of stubble (e.g. by burning) can help to contain the disease. However, the long persistence of inoculum in the soil or on retained stubble, the increasingly common use of low or zero tillage to preserve soils, and environmental concerns against burning practices, leaves breeding and deployment of resistant varieties as a key management strategy (Liu and Ogbonnaya 2015).

Breeding for resistance requires assessing FCR severity to select candidate genotypes. Phenotyping is typically done by quantifying the discolouration or browning of leaf sheath/stems usually as a resistance score (Wildermuth and McNamara 1994; Knight et al. 2012), or by comparing the production of inoculated versus non-inoculated plots (the latter a measure of tolerance to FCR). Because phenotyping is labour intensive, time consuming and can be imprecise, the identification of chromosome regions associated with resistance/tolerance quantitative trait loci (QTL) could help breeding for FCR resistance.

QTL conferring resistance to FCR have been mapped on 13 out of the 21 wheat chromosomes. A comprehensive review was conducted by Liu and Ogbonnaya (2015) and more recently by Kazan and Gardiner (2018). QTL detection has historically been based on biparental populations (DH or RIL) involving well-known resistance sources such as lines *2–49* and *W21MMT70* crossed with susceptible material. Population sizes have been relatively small, ranging mostly between 90 and 150 individuals, with two studies involving populations of about 200 genotypes. While results are not always consistent across studies, which is expected given the different resistance sources and backgrounds, some regions are consistently identified, particularly on chromosomes 3BL, 2DL and 5DS.

While significant progress has been achieved in understanding the genetic determination of resistance to FCR, it has been difficult to transfer that knowledge into breeding. Currently, most of the cultivars grown across the wheat production areas in Australia are classified as susceptible to FCR, with only two cultivars (Sunguard and the recently released LRPB Stealth) showing some degree of resistance or tolerance. Knowledge of the genomic regions conferring resistance to FCR can help to pyramid favourable alleles into elite (i.e. high quality, high yielding and rust resistant) genetic background to improve the germplasm, as demonstrated by Bovill et al. (2010) and Zheng et al. (2017). More recently, efforts have focused on the use of genome-wide association studies (GWAS) to identify regions controlling FCR resistance in varieties and/or breeding materials. Erginbas-Orakci et al. (2018) identified one potentially novel QTL on 3BL from the evaluation of a panel of 126 advanced breeding lines from CIMMYT’s wheat breeding program. Bhatta et al. (2019) evaluated a panel of 125 synthetic hexaploid wheats to study resistance to multiple biotic stresses, including FCR; however, only one of these lines was resistant to FCR, and although significant associations were found for other diseases, no significant association was found for FCR resistance from this GWAS. Jin et al. (2020) using a set of 358 Chinese varieties or breeding lines that have been selected for seedling resistance to FCR, reported significant associations across eight chromosomes of bread wheat, with potentially novel QTL on 5DL for seedling resistance to FCR. Finally Rahman et al. (2020) used GWAS on 475 lines to detect 23 marker trait associations and then successfully used these associations via marker assisted recurrent selection (MARS) to improve FCR in two bread wheat populations, with lines derived after two cycles of MARS showing improved levels of resistance compared to the base population.

In an effort to enhance resistance to FCR and support incorporation of this resistance into released varieties, a breeding scheme was set up in 2010 to combine resistances from multiple loci with elite Australian varieties. A complex crossing scheme involving backcrosses and intercrosses, in combination with various rounds of selection, resulted in a population with improved resistance to FCR. The program aimed to pyramid FCR-resistant QTL from eight donor lines within the genetic background (> 90%) of Australian bread wheat (susceptible) varieties and so included two cycles of backcrossing to the susceptible elite parents. Here, we report the results of QTL mapping from 985 genotypes that were part of this pre-breeding population, using a genome-wide association (GWAS) approach. The population includes founder genotypes (susceptible elite and resistance donors), and selected individuals at different levels of the pedigree structure (intermediate and final). We report 17 regions showing a significant association with FCR resistance, which complement previous findings. By investigating allele frequencies of the significant SNP, we show that the frequency of the favourable alleles for resistance to FCR were increased in the population by phenotypic selection, indicating that the pyramiding process was indeed successful. Further, it should be noted that backcrossing to the susceptible parents, in the absence of selection, should have dramatically reduced the frequencies of the resistant alleles; however, the increased frequencies of the favourable alleles and a strong skew in observed phenotypes to resistance demonstrate the QTL detected control resistance to FCR in this bread wheat population.

## Materials and methods

### Plant material

**Fig. 1:**
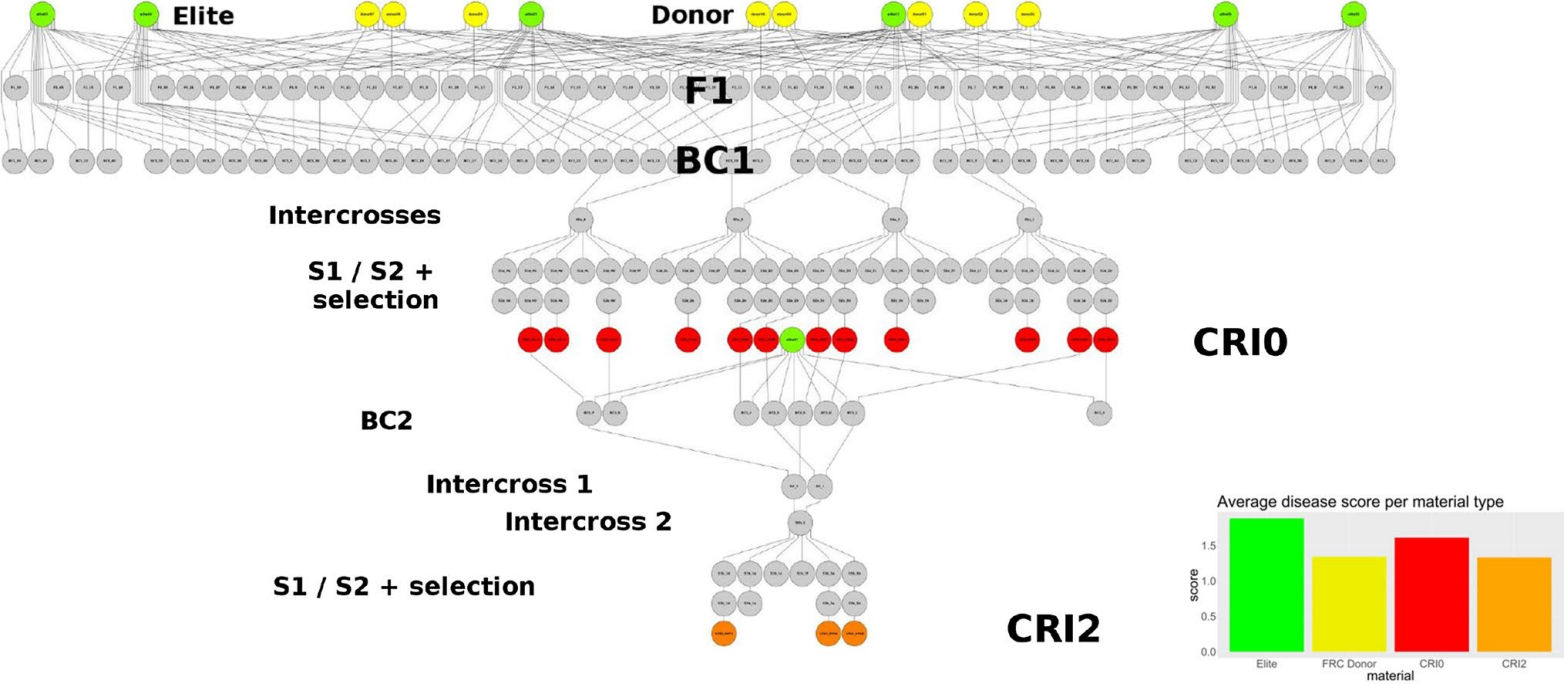
Overview of the pedigree of a population developed to pyramid resistance alleles into Australian elite wheat background. The node’s colours correspond to the type of material: yellow = resistance source (donor), green = susceptible elite, red = intermediate S2:3 lines obtained after backcrossing, intercrossing, selfing, and selection (CRI0), and orange = final S3:4 lines also after further backcrossing, intercrossing, selfing and selection (CRI2). Grey nodes are intermediate stages/generations in the pedigree. Phenotype and SNP data were available for all but the grey nodes. As for illustration purposes, only a small subset of the total population is shown. The bar-plot shows the average disease score per material type

The population used in this study was developed with the objective of pyramiding favourable alleles for FCR resistance from eight ″donors″ (sources of resistance) into an ″elite″ (i.e. high yield, high quality) wheat genetic background represented by eleven commercial varieties in Australia. Development of the population involved crossing, backcrossing and selfing, and included various stages of selection in the segregating generations. A schematic overview of the crossing scheme using the software Helium (Shaw et al. 2014) is shown in Fig. 1.

The population was initiated by crossing eight FCR donor lines with six cultivars generating 48 F1s (Fig. 1). The eight donor lines were sourced from four different programs: E17 (Batavia/Ernie) and L2-94 (Lang/CSCR6) from Chunji Liu (CSIRO); LRC2009-012 (2–49/W21MMT70) and LRC2009-119 (QT10162/W21MMT70) from Damian Herde (Queensland Government); DH#37 from Kukri/Janz and DH#41 from Frame/Sentinel were supplied by Hugh Wallwork (South Australian Government); and AUS33735 (AUS GS50AT34/Sunco//Cunningham) and AUS33802 (SABUF/7/ALTAR 84/AE.SQUARROSA(224)//YACO/6/CROC_1/AE.SQUARROSA(205)/5/BR12*3/4/IAS55*4/CI14123/3/IAS55*4/EG,AUS//IAS55*4/ALD) from the Australian Grains Genebank, both of which were imported into Australia from CIMMYTs soil borne diseases program in Turkey. The initial six elite Australian varieties were EGA Gregory, Ventura, Gladius, Magenta, Scout and LRPB Crusader. Each of the 48 F1s generated was then backcrossed with the corresponding elite variety as a recurrent parent. Then, BC1s with a common recurrent parent were intercrossed with each other. Note, in order to simplify the picture, in Fig. 1 only four of these BC1 x BC1 crosses are shown. The intercrosses (considered the S0 generation) were advanced to S2:3 lines by two cycles of selfing and selection. The S2:3 lines mark the end of the first selection cycle and were labelled as CRI0. Seed of each derived line was multiplied through additional cycles of selfing. Note further that only a few of the CRI0 lines are shown as red circles in Fig. 1 and that not all S0 individuals gave rise to a CRI0 line to highlight selection. The intermediate (i.e. the CRI0and similarly for the final set of CRI2 lines) were primarily selected for resistance to FCR, usually in controlled temperature glasshouses under high inoculum load, with single resistant plants selected in the segregating generations. During seed multiplication phases, fixed lines were evaluated for plant height, maturity and rust resistance. Some lines that were tall, matured late or were susceptible to yellow rust were discarded at this stage. Where possible, selection was also imposed in the segregating generations for height, maturity and rust resistance, because a number of the original FCR donors were tall, late maturity and susceptible to rusts.

From the CRI0 lines, a second cycle of selection was initiated by backcrossing selected CRI0 lines (not all CRI0 lines were backcrossed) with either the same recurrent parent or a similar elite variety. At this stage, five additional varieties entered the crossing scheme (EGA Gregory, Scout and Magenta were retained, while Sunguard, Mace, Emu Rock, LRPB Spitfire and Wallup were added), raising the total number of elite varieties contributing to the pedigree to a total of eleven. Two steps of intercrossing followed the backcrossing (always within the same elite background), resulting in offspring with between six and eight of the original FCR donor lines in their pedigree. After the intercrossing steps, a second round of selfing and selection in the segregating generations followed to produce the S3:4 lines labelled as CRI2 lines in Fig. 1. Similar to the previous cycle of selection, not all the CRI0 lines resulted in an offspring at the bottom of the pedigree, and seed of each line was multiplied through additional cycles of selfing for purposes of phenotyping.

The total population used in this research consisted of 985 genotypes including four of the original eight FCR donors (i.e. L2-94 (Lang/CSCR6), LRC2009-012 (2–49/W21MMT70), DH#37 from Kukri/Janz and DH#41 from Frame/Sentinel were included), nine of the eleven elite cultivars used as parents (Emu Rock and Wallup were not included in phenotyping experiments), 327 CRI0 lines, 642 CRI2 lines, plus W21MMT70 as a resistant check, and Batavia as a susceptible check, and EGA Wylie as best resistant released variety (at commencement of the study). For all genotypes, both phenotypic and molecular marker information was available. Note that an intermediate set of lines (labelled CRI1) were also derived from this program, but are not included here as genotype data were not available for these lines. For simplicity of presentation, later the resistant check W21MMT70 is grouped with the FCR donors, and the released varieties Batavia and Wylie are grouped with the elite parents.

### Phenotyping

The phenotyping for adult plant FCR was obtained from pot trials conducted under controlled conditions (i.e. a temperature controlled glasshouse). Four trials were run to evaluate the CRI0 lines (in 2013 and 2014), and another four trials to evaluate the CRI2 lines (in 2015 and 2016). The trial layout was adjusted to the different facilities and number of lines to be tested and resulted in different layouts (for example, different levels of incomplete blocking structure). The number of replicates per genotype was usually four, but in some trials was larger (six) or smaller (three, and in a few cases genotypes were un-replicated due to a few missing values). In all the trials, a common set of resistant and susceptible controls were included and highly replicated within each trial. Spatial information (row and column coordinates) was available for most trials. The phenotyping methods were refined slightly across the four years of this project, achieving greater uniformity with less unexplained variation.

To assess resistance to FCR, plants were inoculated three weeks after emergence (at 2–3 leaf stage) and then scored at maturity for stem browning on a 0 to 4 scale, with 0 most resistant and 4 most susceptible (Wildermuth and McNamara 1994). In some of the experiments, the scores per experimental unit were the average of several individual plants within the unit. In all cases, a mixture of aggressive FCR isolates were used—isolates were grown on autoclaved grain (wheat), dried and frozen (at − 20 °C) prior to use. The inoculated grain was milled to powder in a commercial coffee grinder, and the different isolates mixed prior to use. A layer of ground whole wheat was added to the top of each pot, and the mixed inoculum placed in the centre of each pot and covered with additional grown wheat. The inoculum rapidly colonized the ground wheat and covered to top of each pot. The high inoculum pressure meant that there were unlikely to be any escapes.

### Genotyping

The population was genotyped with the Illumina iSelect 90,000 SNP bead chip assay as described in Wang et al. (2014). After filtering out monomorphic markers, SNP with rare alleles (minor allele frequency < 1%), and SNP with more than 30% missing values, a final set of 21,365 SNP were retained. For each SNP, allele frequencies were estimated and markers coded as 0, 1, 2 according to the minor alleles count in a particular genotype.

Genetic map positions of the SNP were based on the integrated map from Wang et al. (2014) and resulted in more than 1000 SNP per chromosome in the A and B genomes, and between 97 and 585 SNP per chromosome in the D genome. The median distance between SNP was less than 1 cM in all but two chromosomes (1.03 and 1.41 cM for chromosomes 5D and 7D), and the distance between consecutive SNP was typically smaller than 5 cM, with good marker coverage on all chromosomes.

### Data analysis

A two-stage approach was followed for the phenotypic analysis, where trials were first analyzed individually to obtain best linear unbiased estimates (BLUEs) of genotypic disease scores with corresponding weights (Smith et al. 2001; Mohring and Piepho 2009). The statistical model for each of the trials included fixed and/or random terms to accommodate the experimental design features (blocks taken fixed if complete, random otherwise), a fixed genotype effect (including checks) and spatial modelling (AR1xAR1 processes) applied to the residuals when necessary.

The BLUEs of each genotype per trial were combined in a joint weighted analysis with experiment and genotype as main effects in the model. The resulting adjusted means across trials were used as the phenotypic data in the marker–trait association study (GWAS).

A GWAS study aims at identifying genomic regions (i.e. individual SNP markers or marker haplotypes in this case) that are associated with the target trait. The starting point is a null model where the total genetic variation is modelled by random genotypic effects (structured by some relationships) and a residual (Eq. 1):

1 $$y_{i} = \mu + G_{i} + \varepsilon_{i}$$

where *y*_*i*_is the BLUE across trials for genotype *i*,*µ* is a general intercept, *G*_*i*_a random genotypic effect with variance $\sigma_{G}^{2}$ and structured according to a relationship matrix ***A*** estimated from SNP, and *ε*_*i*_ a residual with constant variance *σ*^2^. The estimation of the relationship matrix ***A*** was performed following van Raden (2008) as implemented in the *kin()* function of the Synbreed R-package (Wimmer et al., 2012). However, instead of estimating a unique relationship matrix based on all the available SNP, we estimated 21 different matrices ***A***_***c***_ with *c* = 1*…*21, using all but the SNP mapping on the particular chromosome. For example, when estimating ***A***_1_, all SNP except those on chromosome 1A were used, and the relationship matrix used in Eq. 1 to define the null model for testing SNP on chromosome 1A. Rincent et al. (2014) showed that using SNP that are in linkage disequilibrium with the tested SNP to estimate the relationship matrix ***A*** can lower the power of the test and therefore the motivation for a chromosome-specific relationship matrix to set up the null model.

The GWAS model is an extension of Eq. 1 with an extra fixed effect *α* associated with the SNP represented by the explanatory covariate *x* (Eq. 2). The possible values of *x*_*i*_∈ (0,1, 2) reflect the allele count of one of the two SNP alleles (arbitrarily chosen). We adopted the common practice of choosing the count of the low-frequency allele (the ″minor″ allele). Therefore, the intercept in model 2 corresponds to the estimate of the mean disease score of the homozygous genotype with the common allele at the SNP, and *α* to the change in disease score when replacing one of the common alleles by the minor allele. Within the context of this research, it means that if *α* < 0, the minor allele is the favourable one, and if *α* > 0, the common allele is the one to select for.

2 $$y_{i} = \mu + \alpha x_{i} + G_{i} + \varepsilon_{i}$$

With the extra fixed SNP effect, the mixed model can be fitted by REML to obtain estimates of variance components, SNP effects and produce a test statistic that accounts for relatedness and so avoid spurious associations (Kennedy et al. 1992; Yu et al. 2006; Malosetti et al. 2007). Because this requires the inversion of rather large and dense matrices, the approach can become impractical for a large number of markers. An alternative is to do the genome-wide scan by generalized least squares (GLS) estimation conditional on the variance–covariance of the data estimated from the model without SNP (Kang et al. 2010; Kruijer et al. 2015). Here, we used the GLS scan that has been implemented in C +  + within the Genstat environment (Boer et al. 2015), which made it possible to very efficiently scan > 21,000 SNP in a population of nearly 1000 individuals.

Significant SNP was identified using a conservative threshold of −*log*_10_(*P*) = 4 (to account for multiple testing). For the significant SNP, the full mixed model was fitted again to obtain final estimates of their effects (although GLS estimates should be adequate, by re-fitting the mixed model the variance–covariance model is conditional on the fitted SNP, which is not the case in the GLS fit). Based on the sign of the effects, at each SNP either the common or the minor allele was tagged as “favourable”, and the frequency of each favourable allele estimated within the different groups in the population: elite, donor, CRI0 and CRI2.

## Results

**Fig. 2:**
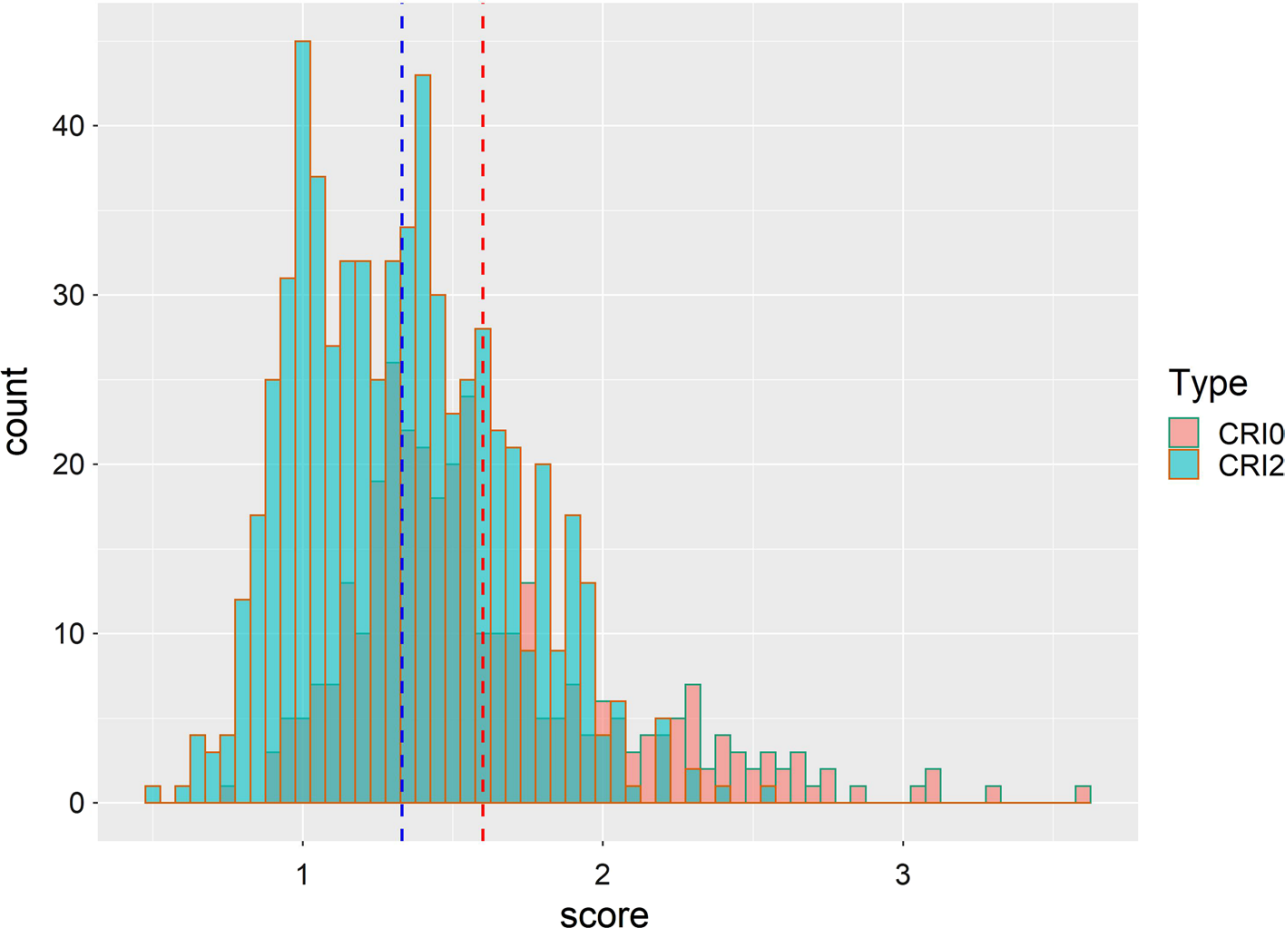
Distribution of the overall mean values for Fusarium crown rot resistance estimated from the CRI0 and CRI2 lines used in the GWAS analysis. Mean values of the CRI0 and CRI2 populations are indicated by vertical red and blue dashed lines, respectively

**Fig. 3:**
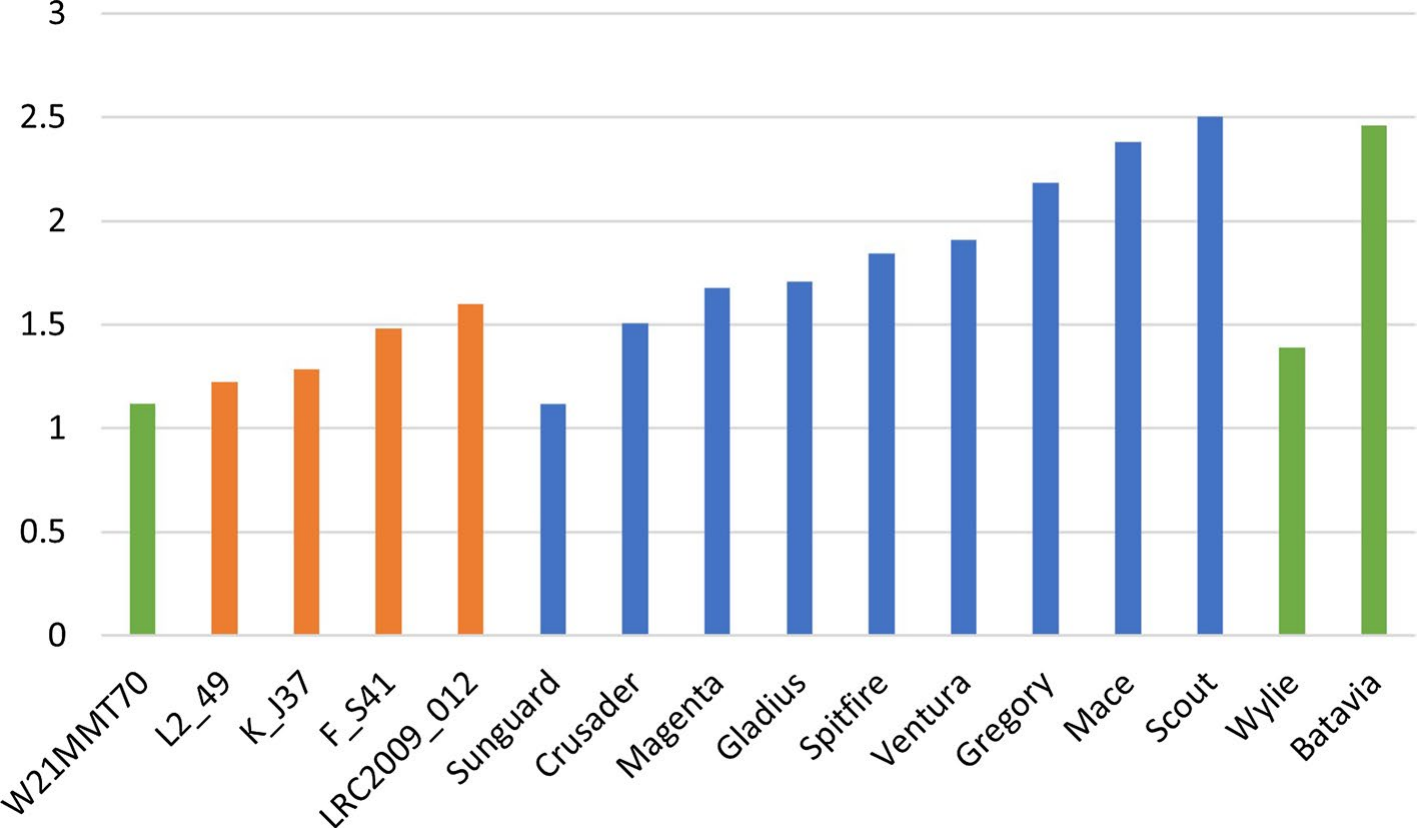
Mean Fusarium crown rot (FCR) resistance score of the donors (orange), elite parents used to produce the CRI0 or CRI2 lines (blue) and resistant or susceptible checks (green) included in the FCR resistance phenotyping experiments from 2013 to 2016

Broad sense heritability estimates for FCR resistance measured in the various experiments used in the analyses reported here, range from a low of 0.19 in one of the early phenotyping experiments to a high of 0.62, averaging 0.38 across experiments. The mean disease scores by material type in the population (Table 1) demonstrates that as expected, the original FCR donors are significantly more resistant than the elite varieties used as parents (i.e. lower stem browning score). Although not significantly different (*α* = 0*.*05), the intermediate generation (CRI0) showed a higher resistance level (lower disease score) than the elite, which suggests a change of resistance in the desired direction. The most advanced, CRI2 generation, showed a significant increase in resistance with respect to the original elite, and the average level of resistance was comparable to that of the original donors. Figure 2 shows the distribution of the overall mean phenotypic values for FCR resistance of all CRI0 and CRI2 lines, demonstrating strong skew towards resistance in this populations, and a large number of derived lines (particularly the CRI2 lines) showing higher levels of resistance than then original FCR donors. The elite varieties used as parents demonstrated a range of response to FCR, with the variety Sunguard showing comparable resistance to some of the FCR donors, and Crusader, Magenta and Gladius showing intermediate resistance relative to the susceptible and resistant check varieties (Fig. 3), These results suggest that the pyramiding of resistant alleles has been successful and was the starting point of this research. The question was to shed more light by investigating the chromosome regions behind the observed response to selection.

**Table 1:** Mean disease resistance score (0 = resistant, 4 = susceptible) of the different types of material in the population: elite, donor, CRI0 and CRI2 genotypes. The number of individuals per group is given in column *N*. Means followed by different letters differ statistically (*α* = 0*.*05). Refer to Fig. 1 for further information on the breeding scheme used

Material	*N*	Mean disease score	Std. error
Elite	11	1.88 a	0.14
FCR donor	5	1.34 b	0.09
CRI0	327	1.61 a	0.03
CRI2	642	1.33 b	0.01

**Fig. 4:**
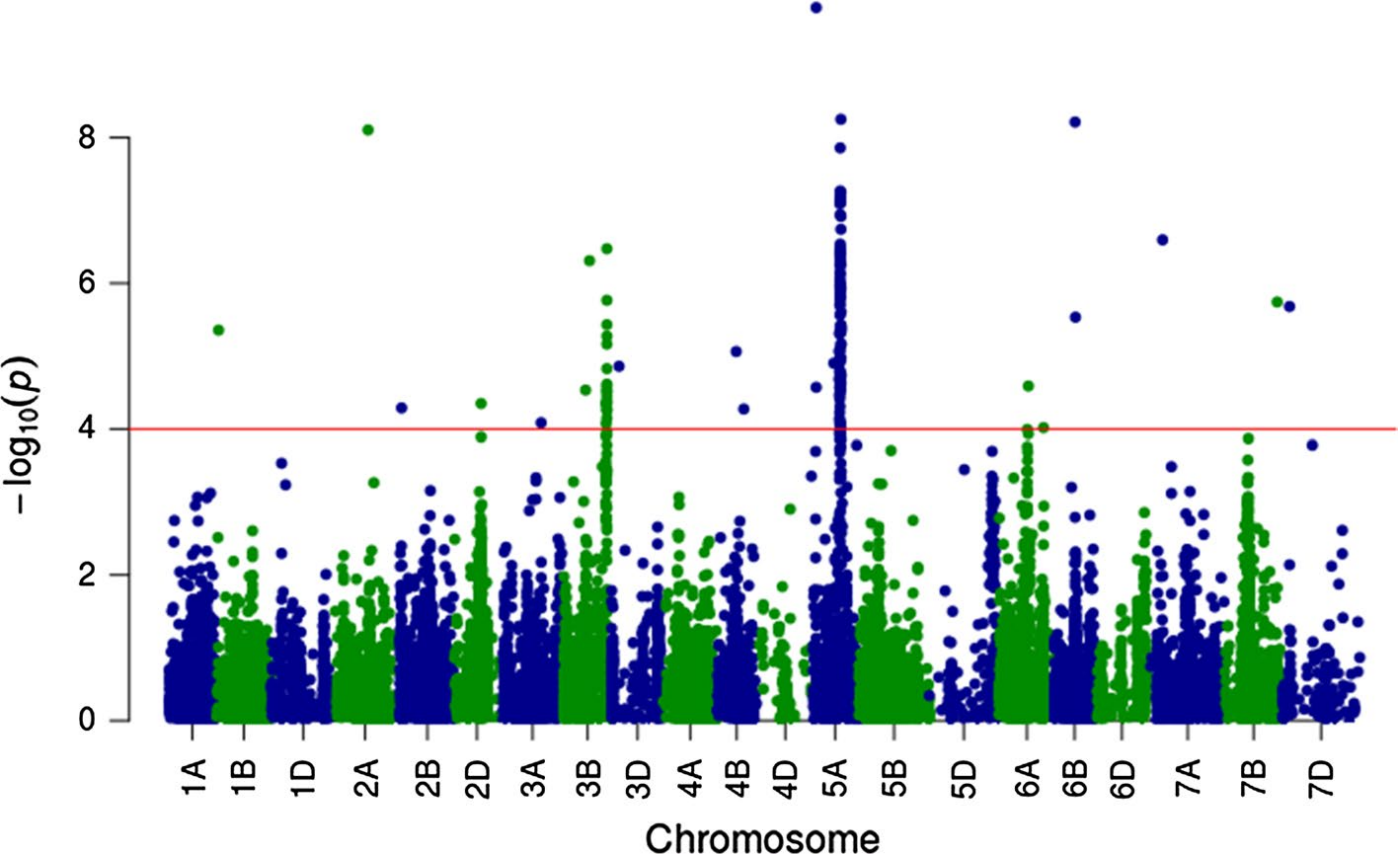
Manhattan plot showing the strength of the association with the FCR disease score (on a −*log*_10_(*P*) scale versus the SNP position ( 21 K SNP across 21 chromosomes sorted by number and genome A, B and D). The horizontal red line shows the significance threshold (−*log*_10_(*P*) = 4)

Assessment of 21,365 SNP for significant association with the disease score is shown in Fig. 4. While the plot highlights two regions (one on chromosome 3B and another on chromosome 5A), 126 SNP were found above the threshold on 14 different chromosomes (1B, 2A, 2B, 2D, 3A, 3B, 3D, 4B, 5A, 6A, 6B, 7A, 7B and 7D). When taking a minimum gap of 30 cM between SNP to consider them as tagging different QTL, a total of 17 chromosome regions (QTL) were found to be significantly associated with the observed FCR disease resistance scores.

**Fig. 5:**
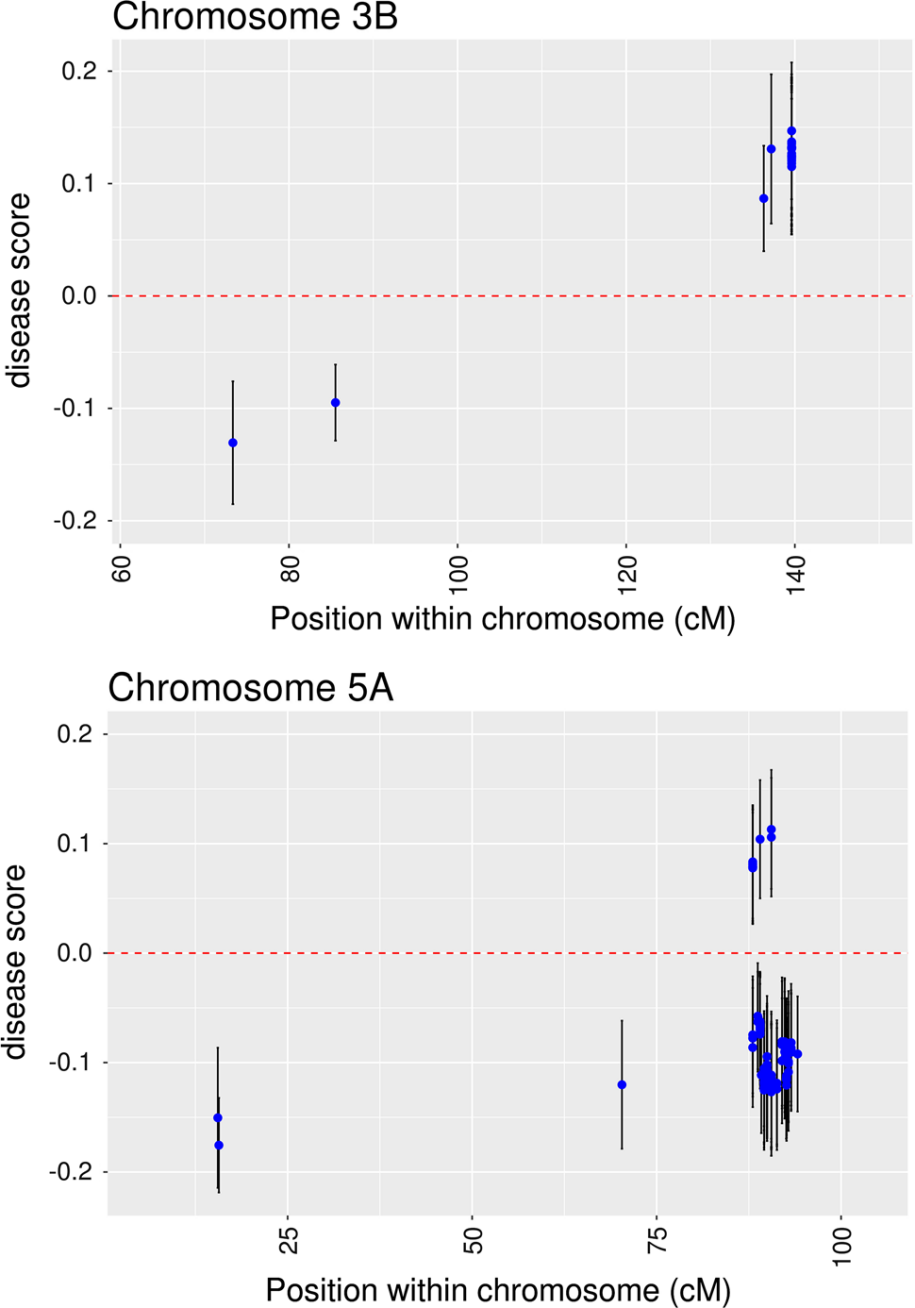
Point estimates (and 95% confidence interval) of the minor SNP allele effect at the significant SNP found on chromosomes 3B and 5B. The horizontal axis indicates the SNP position on the corresponding chromosome

For each of the 126 SNP significantly associated with FCR resistance, the full mixed model was fitted (Eq. 2) to obtain SNP allele effects (and standard errors). To illustrate, the point estimates and confidence intervals of the SNP minor allele effects on chromosome 3B and 5A are shown in Fig. 5.

On chromosome 3B, SNP can be grouped in two regions (QTL): one around 80 cM and a second region around 140 cM (Fig. 5). The point estimate of the effects of the SNP around 80 cM was − 0.1 to − 0.13, meaning that the minor allele was associated with a lower score. The opposite was true for the SNP located around 140 cM, with the effect positive, pointing to the common allele as the one associated with a lower disease score. On chromosome 5A, SNP can also be grouped in two main regions. A set of SNP around 15 cM show a relatively strong effect (− 0.15 to − 0.17), pointing to the rare allele as the one in coupling with the favourable allele. A large group of SNP appear within the region 88–94 cM and an extra SNP at 70 cM. This latter SNP is most likely associated with the same QTL. In this region, the signs of the effects of the minor allele were not always the same. For most of the SNP, the sign was negative, pointing to the minor allele in coupling with the favourable QTL allele. However, in some cases the sign was positive, suggesting the common allele is associated with the favourable QTL allele. This apparent contradiction can be clarified when inspecting the SNP allele frequencies. We observed that for most of the SNP within this region, the minor allele occurred at relatively high-frequency values (above 0.45), which can explain the reversals in sign by simple changes of the arbitrary reference level. We should emphasize here that we are referring to the SNP allele frequencies and not the underlying causative QTL alleles (which were unobserved). Therefore, the changes in sign reflect reversals of the linkage phase (coupling/repulsion) between SNP minor alleles and the favourable QTL allele.

**Fig. 6:**
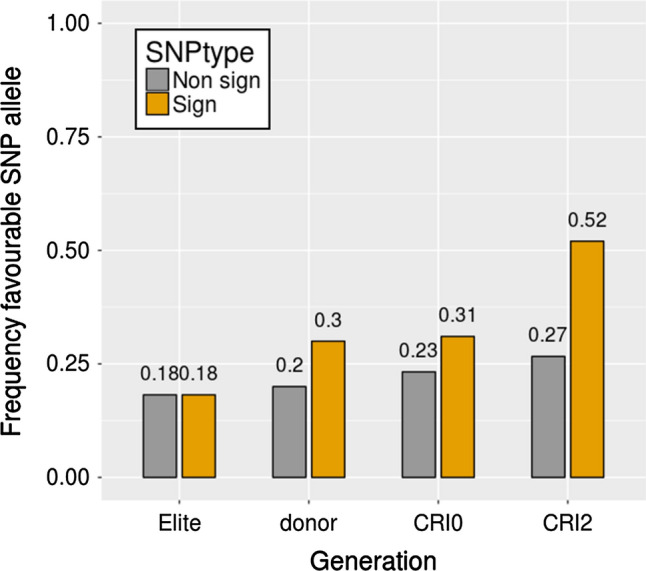
Frequency of the “favourable” SNP allele at 126 SNP loci significantly associated with FCR resistance. The grey bars show minor allele frequencies of 500 randomly chosen SNP that were not associated with FCR resistance

**Fig. 7:**
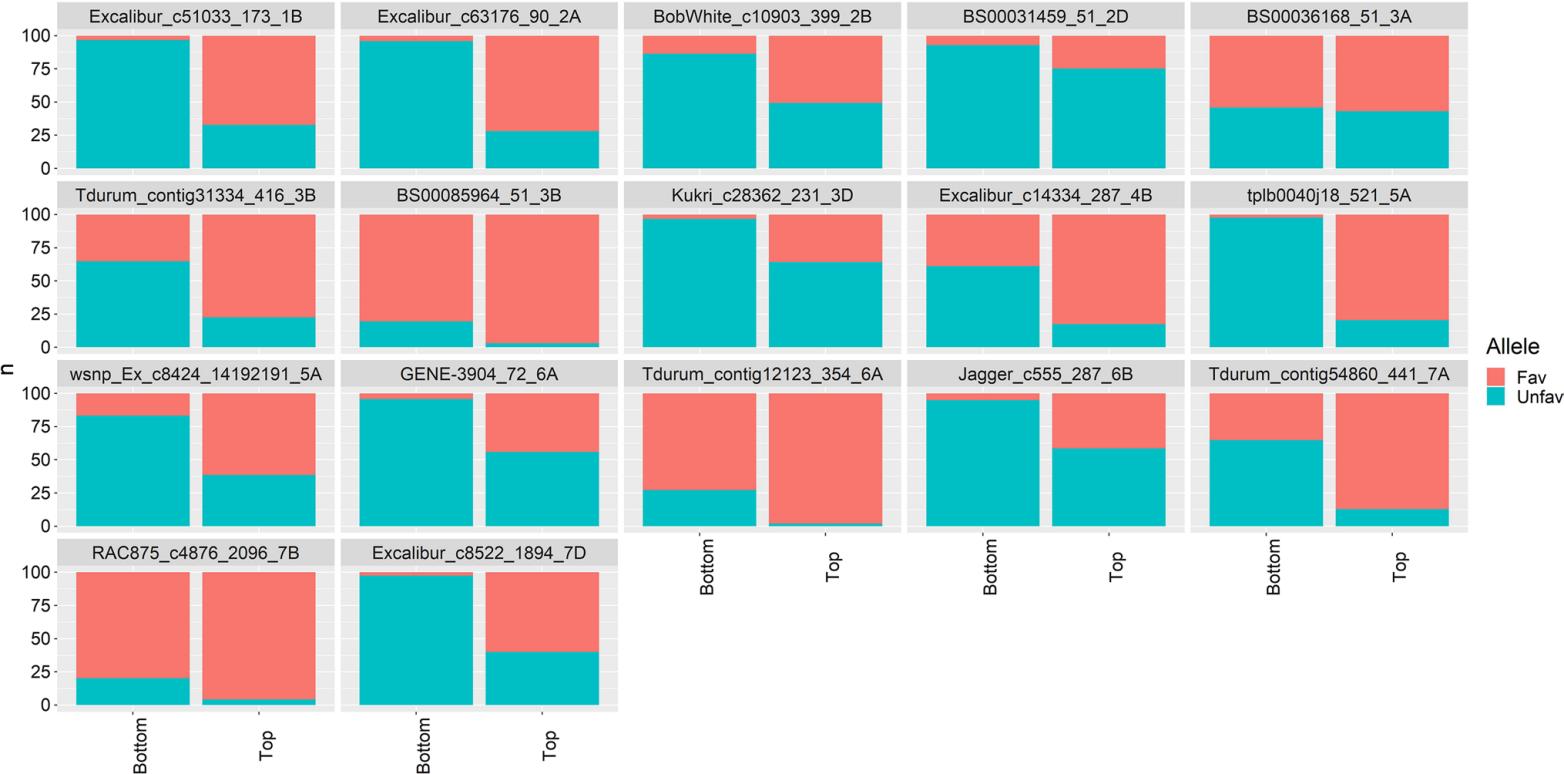
Percentage of the “favourable” and “unfavourable” alleles as each of the 17 SNP loci identified as being significantly associated with Fusarium crown rot (FCR) resistance, in the 100 lines (including parents) with the best (i.e. lowest) FCR resistance mean values (i.e. ″Top″) and the worst (i.e. highest) mean FCR resistance values (i.e. ″Bottom″)

Although indirectly, changes in the alleles frequencies at the significant SNP can reflect changes of QTL alleles frequencies due to selection. The assumption is that “favourable” SNP alleles (indicated within quotes, as the SNP alleles are not necessarily causative) are in coupling phase with the causative (unobserved) favourable QTL allele. The average frequencies of the “favourable” SNP allele at the significant SNP (aggregated by genetic material) are shown in Fig. 6. For reference, the mean frequency of the minor allele of 500 randomly sampled SNP that were not associated with the disease score is also shown in the plot. The frequencies of the alleles of the neutral SNP (grey bars in Fig. 6) did not show changes between groups (i.e. the elite parents, CRI0, CRI2 and FCR donors). However, a clear trend was observed for the 126 SNP that were significantly associated with FCR resistance, with the frequency of the favourable SNP alleles the lowest in the elite material, and the highest in the set of most advanced material (CRI2). This trend suggests that the regions tagged by SNP detected in the GWAS analysis are linked to chromosome regions under selection pressure. To explore these changes in gene frequency further, the frequency of the favourable alleles for each of the 17 SNP loci most strongly associated with each QTL was compared between the ″Top″ and ″Bottom″ genotypes out of the nearly 900 genotypes assessed in this study (Fig. 7). The ″Top″ group included the 100 genotypes (lines or parents) with the best (i.e. lowest) resistance scores with an average score of 0.9 and values ranging between 0.5 and 1.0. The “Bottom” group included the 100 genotypes with the worst (i.e. highest) resistance score with an average score of 2.3 and ranging between 1.9 and 3.6. For some loci (e.g. BS00065964_51 on 3B, Tdurum_contig31334_416 on 3B and Tdurum_contig12123_354 on 6A), the frequency of the favourable allele is quite high in this population), while for others the frequency of the favourable allele is quite low (e.g. BS00031459_51 on 2D). Nevertheless, an increase in the favourable allele was observed at each of these 17 loci in the ″Top″ compared to the ″Bottom″ 100 genotypes. The shift of allele frequencies towards an increase of the favourable QTL alleles is consistent with a successful resistance introgression program, particularly when backcrossing to the susceptible elite parents, will result in reduction in the frequency of favourable QTL otherwise.

## Discussion

Phenotypic and genotypic data were obtained for a diverse population in a pre-breeding program involving multiple crossings and cycles of selection. As a result of selection, the population showed a significant increase in resistance, with the distribution of mean disease resistance scores strongly skewed towards resistance, supporting the success of the phenotypic (conventional) breeding process. Had selection not been effective, backcrossing to the elite (susceptible) parent would be expected to reduce the level of resistance in the derived populations. This contrasts with distributions for FCR resistance observed in unselected populations (e.g. Erginbas-Orakci et al. 2018 where observed adult resistance is highly skewed towards susceptibility, and Jin et al. 2020 where disease index scores for seedling resistance was approximately normally distributed). A total of 17 chromosome regions (QTL) were identified, each conferring some resistance to FCR in bread wheat. In this respect, the results are in agreement with the existing literature pointing to the quantitative nature of resistance, with many genomic regions associated with resistance to FCR, as has been previously reported by a number of authors (Jin et al. 2020; Rahman et al. 2020; Zheng et al. 2014; Poole et al. 2012; Collard et al. 2005, 2006; Bovill et al. 2006). However, there are important differences between the study here and previous ones that have consequences on the interpretation and possible impact of the reported findings.

Firstly, the current study utilized a population of almost 1000 individuals, 5–10 times larger than most previous studies, and more than double the size of recently published GWAS by Jin et al (2020) and Rahman et al. (2020). Additionally, many previous studies for QTL mapping of FCR resistance have relied upon biparental populations (double haploids and recombinant inbred lines), whereas the population used here involved the crossings of several susceptible and resistant founders, akin to a multi-parental population such as MAGIC (Cavanagh et al. 2008). With the exception of the work published by Rahman et al. (2020), this is the only study that has been able to track the effect of selection on changes in the frequency of the favourable alleles, demonstrating positive change in the resistant phenotype from selection. Further, in contrast to standard biparental or multi-parental experimental mapping populations, this study utilized a population from a current pre-breeding project, allowing selection to shape population structure. The components of the population were not part of a single stratum of the pedigree as it is usual in mapping populations, but spanned several layers (founders, intermediate and final offspring). This gives an implicit time-dimension in the data that can shed light on the trait and marker allelic dynamics across the population history.

The advantage of increased population size is clear, as larger populations should allow QTL to be detected more easily and precisely (Weller and Soller 2004). Involving multiple parental lines in the crossing scheme can increase the chances to find QTL as the higher allelic diversity increases the odds of segregating loci (Xu 1998). The use of genotypes that are part of a breeding population has at least two benefits. The first is one of convenience because no additional experimental population has to be set in parallel to the breeding population. The second is one of relevance because the QTL are detected and their effects estimated within a relevant background, hopefully shortening the gap between knowledge generation and application. Although a generally recognized limitation of many QTL mapping efforts, the large gap between QTL detection and actual use in breeding has been highlighted in the area of FCR resistance research (Liu and Ogbonnaya 2015).

Using a breeding population instead of an experimental population in QTL detection presents its difficulties as well. Because selection is an important factor in any breeding program, precautions should be taken to avoid false positives caused by linkage disequilibrium stemming from pedigree relationships (Malosetti et al. 2011). This was the main motivation for applying a classical GWAS approach in this study. While there exists more elaborate approaches (addressed later), here we argue for a simple first approach that can quickly generate results of immediate use to breeders.

The GWAS analyses identified 17 different QTL, a large proportion of which have been reported previously (Liu and Ogbonnaya 2015), and many most probably the same as the 23 QTL identified by Rahman et al. (2020) but with some notable differences. For example, a clear indication was observed on the long arm of chromosome 3B, a region consistently reported to harbour a QTL for FCR resistance (Rahman et al. 2020; Ma et al. 2010; Li et al. 2010; Poole et al. 2012) but absent from the study of seedling resistance by Jin et al. (2020) in Chinese germplasm. This QTL has been detected in multiple genetic backgrounds and with different sources of favourable alleles including a *T. spelta* source called CSCR6, a relatively old breeding line (IRN497), a synthetic hexaploid (CPI133814), and a number of varieties (Ernie, Macon, Otis, and Sunco) (Liu and Ogbonnaya 2015), many of which are represented in the pedigree of the FCR donors used in the study reported here. In addition, this QTL has been fine-mapped using near isogenic lines derived from crosses with CSCR6 (Zheng et al. 2015b). Other recurrent QTL in the literature occurs on the long arm of chromosome 2D and on the short arm of chromosome 5D. While we did detect a QTL on 2D, the signal was not strong enough on chromosome 5D (although we did observe a signal with −*log*10(*P*) > 3).

We also found QTL on chromosomes where no QTL has been previously reported, notably between 88 and 94 cM on chromosome 5AL which was only recently reported by Jin et al. (2020), but was absent in study reported by Rahman et al. (2020). A large number of SNP in this region were shown to be in LD with an FCR resistance allele. Upon inspection of SNP haplotypes in this region using the graphical genotype visualization software *Flapjack* (Milne et al., 2010), one of the few possible sources of the favourable SNP allele seemed to be a donor line derived from the cross between cultivars Kukri and Janz. The Kukri/Janz cross was originally made by Kammholz et al. (2001) to establish a double haploid mapping population that combined good dough quality traits (Kukri) with wide adaptation and high yielding (Janz). In addition, the population segregated for several disease resistance alleles (Kammholz et al. 2001), and both Kukri and Janz have been shown to contribute resistance alleles to FCR (Wallwork et al. 2004; Martin et al. 2015). Two other possible sources of the favourable SNP allele were cultivars Gladius and Sunguard. The latter contains Janz in its pedigree and is described as resistant to FCR (at a similar level as cultivar Wylie and is considered as the current FCR-resistant reference in Australia). These results suggest that this region on chromosome 5A could be important and deserves future attention.

While the time dimension in the data (early versus late generations) did not play a role in the QTL detection, we did observe a clear trend in the allele frequencies at the significant SNP as a function of time. The SNP alleles that were positively correlated with a higher resistance to FCR, were the ones that increased in frequency when moving from earlier to later generations in the pedigree. This contrasted to the lack of trend that was observed in the allele frequencies of a random sample of SNP that were not significantly associated with FCR resistance (i.e. selectively neutral). This gives additional support to the findings here, as the significant SNP must be in LD with QTL alleles behind resistance to FCR. This is an advantage of having used a breeding population with a known selection history, in contrast to a classical GWAS panel where the selection forces are unknown.

We used a marker–trait association approach similar to many GWAS studies. A clear advantage of the approach is its simplicity from a statistical modelling point of view, which is probably one of the reasons that makes GWAS so popular. For each of the (many) markers, individuals in the population are classified by their allele status and the groups are contrasted (usually two groups, since most markers are SNP). The analysis boils down to an ANOVA or a t-test with the additional complication that residuals show various degrees of dependency, and so, some covariance modelling is required. The modelling of the residual has to be done based on estimated background relationships (Kennedy et al. 1992). Modern applications typically use genome-wide markers to estimate realized relationships between individuals in the population (van Raden 2008). While obvious, it has not been emphasized enough in the literature that the simplicity of the approach comes along with a cost. The penalty is that QTL detection relies on SNP marker Identity by State information (IBS) as opposed to QTL allele information. SNP and QTL alleles are only interchangeable terms in the special case when the marker allele is a causal variant, which is unlikely to be the most common situation.

The disadvantages of marker-based QTL mapping were earlier recognized in the literature, most notably in the seminal paper by Lander and Botstein (1989). The advantage of approaches that use markers to infer Identity by Descent information (IBD) at the QTL position is clear and has been at the core of linkage analysis by interval mapping. Inferring IBD information typically relies on hidden Markov chain models to estimate conditional QTL probabilities given observed marker information, a genetic map, and a number of population-related assumptions (Jiang and Zeng 1995; Lander and Green 1987). While a powerful approach, especially during the era of low-density markers, the required assumptions regarding the population structure limit their application to relatively simple biparental populations such as F2, recombinant inbred lines, or doubled haploid populations.

The popularization of multi-parental populations for QTL mapping, such as nested association mapping (NAM) (McMullen et al. 2009), the *Arabidopsis* multi-parent advanced generation intercross (AMPRIL) (Huang et al. 2011), or multi-advanced generation intercross (MAGIC) (Cavanagh et al. 2008), has resulted in approaches that can handle more complex populations (Mott et al. 2000; Liu et al. 2010). However, those methods tend to be population specific and are not easily generalized to any type of population. A possible recent exception is a Bayesian hidden Markov model approach published by Zheng et al. (2015a), which can reconstruct ancestry blocks at any point on the genome for any type of population, as long as the pedigree information is available. Software called RABBIT (Reconstructing Ancestry Blocks BIT by bit) can be used to implement the rather mathematical approach. Although not many, there are early examples showing the advantages of an IBD-based QTL detection approach in breeding populations (Crepieux et al. 2005; van Eeuwijk et al. 2010). It will be interesting to compare the performance of the approach used here with a more elaborate IBD-based one, which is matter of current research.

In the title, we suggest that we can learn from a simple GWAS approach applied to a breeding population. For FCR, the most likely genomic regions in wheat were identified that are responsible for the observed improved resistance, and a list of SNP and the corresponding favourable alleles were produced that can potentially be used as proxies to select superior material. At a time in which genomic selection seems to be the only feasible selection strategy, we believe there is still value in identifying the chromosome regions driving the response to selection for particular traits. With this information at hand, breeders can make improved decisions, not only in the selection of material for future crosses, but also in terms of how to combine them within the crossing scheme (e.g. phenotypic and genotypic data can be used to identify resistant lines with complementary QTL, that could then be combined efficiently through marker-assisted recurrent selection (e.g. Rahman et al. 2020)).
